# Neuromuscular electrical stimulation for cancer pain in children with osteosarcoma: A protocol of systematic review

**DOI:** 10.1097/MD.0000000000021311

**Published:** 2020-07-24

**Authors:** Tian-Shu Wang, Shou-Feng Wang, Wei-dong Song, Zhao-chen Tang, Wei Wei, Guan-kai Wang

**Affiliations:** a Second Ward of Orthopedis Department; b First Ward of Orthopedis Department, First Affiliated Hospital of Jiamusi University; c Department of Orthopedics, Second Affiliated Hospital of Mudanjiang Medical University, Mudanjiang; d School of Clinical Medicine, Jiamusi University; e Department of Orthopedics, Graduate School of Jiamusi University, Jiamusi, China.

**Keywords:** cancer pain, effectiveness, neuromuscular electrical stimulation, osteosarcoma

## Abstract

**Background:**

This systematic review will assess the effectiveness and safety neuromuscular electrical stimulation (NMES) for cancer pain (CP) in children with osteosarcoma.

**Methods:**

This systematic review protocol will retrieve the following electronic databases from inception to June 1 in Cochrane Library, MEDLINE, EMBASE, Web of Science, Scopus, CNKI, and VIP database. Manual head-searching of reference lists and conference proceedings will be performed to further examine the articles of interest. No restrictions will be applied to language and publication status. We will utilize a 3-stage approach to scan titles, abstracts, and full-text studies against all eligibility criteria, and collect data from included trials. Study quality will be evaluated by the Cochrane Risk of Bias Tool. If possible, we will narratively summarize study results and carry out meta-analysis.

**Results:**

This study will recapitulate the present high quality trials to appraise the effectiveness and safety of NMES for CP in children with osteosarcoma.

**Conclusion:**

The findings of this study will present evidence to determine whether NMES is effective and safe for CP in children with osteosarcoma.

## 1 Introduction

Osteosarcoma is a very common pleomorphic tumor among pediatric and adolescent population,^[1–3]^ which accounts for about 2.4% of all pediatric cancers.^[1]^ It is characterized by the presence of malignant mesenchymal cells produced in any bone stroma, especially in the long bones, including arms and legs.^[4–6]^ It is often manifests as localized bone pain and swelling.^[7–9]^ It has been estimated that the incidence of osteosarcoma is 2 to 3 cases/million/y among general population.^[10,11]^ However, its annual incidence varies 8 to 11 individuals/million/y in children and adolescents.^[10,11]^ Although the quality of life in patients with osteosarcoma has significantly enhanced over the past few decades, its etiology is still unclear.^[12–14]^ Previous studies found that several multiple factors may be responsible for this disorder, including genetics, epidemiology, and environment.^[15]^

Studies suggested that neuromuscular electrical stimulation (NMES) is utilized to treat cancer pain (CP) in children with osteosarcoma.^[16–18]^ However, no systematic review has explored its effectiveness and safety for CP in children with osteosarcoma. Thus, this systematic review will firstly investigate the effectiveness and safety of NMES for CP in children with osteosarcoma.

## 2 Methods

### 2.1 Study registration

This systematic review protocol was registered on INPLASY202060054. It is designed based on the guidelines of the Preferred Reporting Items for Systematic Reviews and Meta-Analysis Protocol Statement.^[19,20]^

### 2.2 Eligibility criteria

#### 2.2.1 Types of studies

We will include randomized controlled trials (RCTs) that assessed the effectiveness and safety of NMES for CP in children with osteosarcoma. We will exclude other studies, such as non-clinical trial, uncontrolled trials, and non-RCTs.

#### 2.2.2 Types of interventions

Experimental group: all patients received any types of NMES.

Control group: all patients received any interventions, but not any forms of NMES.

#### 2.2.3 Types of patients

Participants (under 18 years old) with confirmed CP in children with osteosarcoma will be included without restrictions to ethnicity, sex, and characteristics of osteosarcoma.

#### 2.2.4 Types of outcome measurements

The primary outcome is pain intensity, as assessed by any pain scales in the reported trials.

The secondary outcomes are frequency of rescue analgesic utilization, cumulative anesthetic drug administration, quality of life, and adverse events.

### 2.3 Data sources and search

The following electronic databases will be systematically retrieved from inception to June 1 in Cochrane Library, MEDLINE, EMBASE, Web of Science, Scopus, CNKI, and VIP database. We will also carry out manual head-searching of reference lists and conference proceedings to avoid missing potential articles. The search strategy will not restrict to any language and publication status. The proposed MEDLINE search strategy with details is created (Table 1). The similar search strategy will be adapted to the other electronic databases. The search strategy will be carried out in conjunction with a research librarian who is an expert in systematic reviews. Additionally, we will carry out head-searching of reference lists and conference proceedings.

**Table 1:**
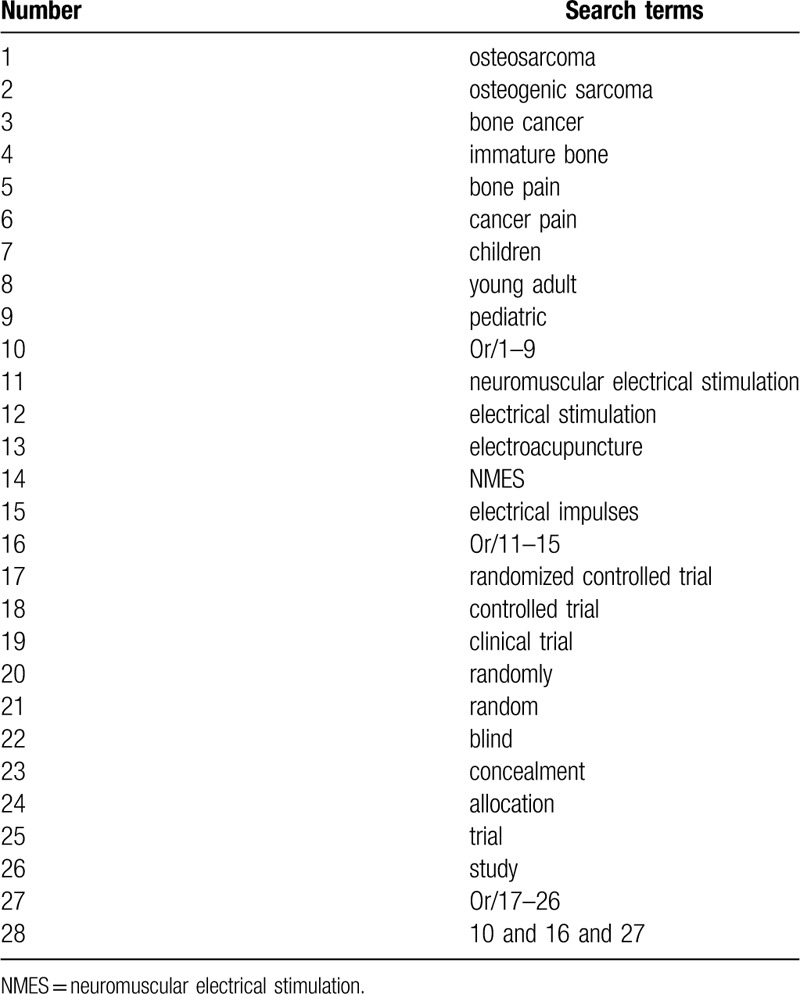
Search strategy for MEDLINE.

### 2.4 Data collection and analysis

#### 2.4.1 Study selection

All searched citations will be managed by Endnote X7, and we will exclude all duplicates. Two authors will independently check titles and abstracts of all records, and we will remove all irrelevant ones. Then, we will read full-text of potential articles to further determine whether they fulfill all eligibility criteria. During the study selection, rationale for all excluded studies will be recorded. Any divergences will be solved by a senior author for reconciliation, and a final conclusion will be reached. A flow chart will be developed to exert the process of study selection at different stages.

#### 2.4.2 Data collection

Two authors will independently collect data using a previously defined data collection form. Any disagreements will be solved by a third author through discussion. The collected data include descriptive information (e.g., study reference, study objective, trial design, title, first author, and geographic location), study population (e.g., diagnostic criteria, inclusion and exclusion criteria, demographic characteristics, and sample size), study methods (e.g., randomization details, blind, and concealment), intervention details (e.g., dosage, duration, and deliver methods), outcome indicators, follow-up information, study results, findings, and conflict of interest.

#### 2.4.3 Missing data dealing with

Whenever insufficient or missing data exists, we will contact original authors to obtain that. If such data cannot be obtained, we will carry out data analysis based on the available data collected from included trials.

#### 2.4.4 Risk of bias assessment

Two authors will independently examine risk of bias using Cochrane Risk of Bias Tool.^[21]^ It covers 7 aspects, and each one is divided into 3 levels: low risk of bias, unclear risk of bias, and high risk of bias. Any confusion will be cleared up by a third author through discussion.

#### 2.4.5 Subgroup analysis

Subgroup analysis will be carried out to check the possible sources that may cause significant heterogeneity according to the different study information, participant characteristics, details of intervention and control, and study quality.

#### 2.4.6 Sensitivity analysis

Sensitivity analysis will be performed to test the robustness of study findings by removing low quality studies.

#### 2.4.7 Reporting bias

Reporting bias will be undertaken using funnel plot and Egger regression test when over 10 eligible trials are included.^[22,23]^

### 2.5 Data synthesis

We will carry out RevMan V.5.3 software (Cochrane Community, London, UK) using statistical analysis. All continuous data will be estimated as mean difference (MD) or standardized MD with 95% confidence intervals (CIs). All dichotomous data will be estimated as risk ratio with 95% CIs. Statistical heterogeneity will be examined using *I*^2^ statistics. It is defined as follows: *I*^2^ ≤ 50% exerts acceptable heterogeneity, and we will use a fixed-effect model. *I*^2^ > 50% means significant heterogeneity, and we will utilize a random-effect model. Meta-analysis will be conducted when the eligible trials are sufficiently homogenous in terms of study design, patient characteristics, details of interventions and controls, and outcome indicators. If meta-analysis is inappropriate, we will report study results by descriptive analysis.

#### 2.5.1 Quality of evidence

Two authors will examine quality of evidence for each outcome using Grading of Recommendations Assessment, Development and Evaluation.^[24]^ Any conflicts will be resolved by a third author through consultation.

### 2.6 Dissemination and ethics

The results of this study will be published through a peer-reviewed journal. This study will not obtain individual participant data, thus, no ethic approval is required.

## 3 Discussion

Osteosarcoma is a very common cancer in pediatric population.^[1–3]^ It accompanies a severe CP in such patients. Previous studies suggested that NMES is utilized for CP in children with osteosarcoma. However, no systematic review investigated the effectiveness and safety of NMES for CP in children with osteosarcoma. Thus, the present systematic review will firstly explore this topic. The findings of this study will summarize high quality trials to assess the effectiveness and safety of NMES for CP in children with osteosarcoma, which may benefit both clinical practice and future research.

## Author contributions

**Conceptualization:** Tian-shu Wang, Shou-feng Wang, Wei Wei, Guan-kai Wang.

**Data curation:** Tian-shu Wang, Zhao-chen Tang, Wei Wei.

**Formal analysis:** Tian-shu Wang, Shou-feng Wang, Wei-dong Song, Wei Wei, Guan-kai Wang.

**Investigation:** Wei Wei.

**Methodology:** Tian-shu Wang, Shou-feng Wang, Zhao-chen Tang, Guan-kai Wang.

**Project administration:** Wei Wei.

**Resources:** Tian-shu Wang, Shou-feng Wang, Wei-dong Song, Zhao-chen Tang, Guan-kai Wang.

**Software:** Tian-shu Wang, Shou-feng Wang, Wei-dong Song, Zhao-chen Tang, Guan-kai Wang.

**Supervision:** Wei Wei.

**Validation:** Tian-shu Wang, Shou-feng Wang, Zhao-chen Tang, Wei Wei, Guan-kai Wang.

**Visualization:** Tian-shu Wang, Wei-dong Song, Wei Wei, Guan-kai Wang.

**Writing – original draft:** Tian-shu Wang, Shou-feng Wang, Wei-dong Song, Zhao-chen Tang, Wei Wei.

**Writing – review & editing:** Tian-shu Wang, Shou-feng Wang, Wei Wei, Guan-kai Wang.
